# O1.4. CEREBROSPINAL FLUID FINDINGS IN TWINS WITH PSYCHOTIC SYMPTOMS – NOVEL FINDINGS AND FUTURE PROSPECTS

**DOI:** 10.1093/schbul/sby015.186

**Published:** 2018-04-01

**Authors:** Viktoria Johansson

**Affiliations:** Karolinska Institutet

## Abstract

**Background:**

Schizophrenia and bipolar disorder are severe mental disorders with unknown etiology. Our research group has studied biomarkers in the cerebrospinal fluid (CSF) of twins with schizophrenia and bipolar disorder to be able to determine the genetic and environmental influences. In brain disorders, CSF is the most appropriate substrate to study as it may reflect the brain biochemistry better than blood. In this presentation I aim to give an overview of our findings and their relation to psychotic disorders. I intend to present our most recent preliminary finding and to discuss future prospects.

**Methods:**

We studied CSF-markers from a cohort of 50 monozygotic (MZ) and dizygotic (DZ) twins with schizophrenia or bipolar disorder. The twins have gone through diagnostic assessments and have been extensively phenotyped with questionnaires, symptom scales for psychiatric symptoms as well as neuropsychological testing. We have analyzed monoamines, microglia-, neurodegenerative-, kynurenine-, and inflammatory markers using immunoassays and high-performance liquid chromatography techniques. We have also studied microscopic structures with scanning electron microscopy.

**Results:**

One of our main findings was that soluble cluster of differentiation 14 protein (sCD14) was higher in twins with schizophrenia or bipolar disorder compared to their not affected co-twins. A later analysis showed that the difference within the discordant twin-pairs was higher in the DZ twin pairs (β=28697.1, t=3.20, p=0.024) compared with the MZ twin pairs (β=5577.5, t=2.10, p=0.081) suggesting that genetic components along with unique environmental effects have an influence on the higher sCD14 levels in patients with schizophrenia and bipolar disorder. We also found that sCD14 was higher in those patients with more psychotic symptoms.

In our study on microscopic structures in CSF we found that the structures were prevalent not only in the patients with schizophrenia and bipolar disorder but also in their not affected co-twins. The finding suggests that genetic factors may be partly involved in the formation of the structures.

**Discussion:**

We have analyzed inflammatory and neurodegerative markers in the CSF of twins with psychotic disorders to be able to study genetic and environmental influences. Our results indicate that sCD14 may have an influence on microglia activation in psychosis. We have continued with analyses on the correlations between all the markers, the monoamine metabolites and associations with symptoms and cognitive ability and the preliminary results from these analyses will be presented.

To conclude CSF analyses for biomarkers in twins may result in extended knowledge regarding the genetic and environmental relationships. Our unique twin data gives us the possibility to study CSF-markers in relation to psychiatric symptoms and cognitive measures. For future studies it would be of interest to assemble twin-samples from several research groups to be able to study research questions regarding gene and environment interactions.

